# Supplementary material for: Evolving Southern Ocean overturning in warming climates
Source: Nat Commun. 2025 Nov 25;16:10449. doi: 10.1038/s41467-025-65389-5 (PMC12647794; doi:10.1038/s41467-025-65389-5)
Supplement: Supplementary file 1 — Supplementary Information [file 41467_2025_65389_MOESM1_ESM.pdf]

Supplementary Information for

Evolving Southern Ocean overturning in warming climates

Tingting Zhu[1*], Wei Liu[1]

[1]Department of Earth and Planetary Sciences, University of California, Riverside, CA, USA

*Corresponding author. Email: tzhu@ucr.edu

This file includes:
Supplementary Figures 1-5
Supplementary Tables 1-2

Supplementary Figures

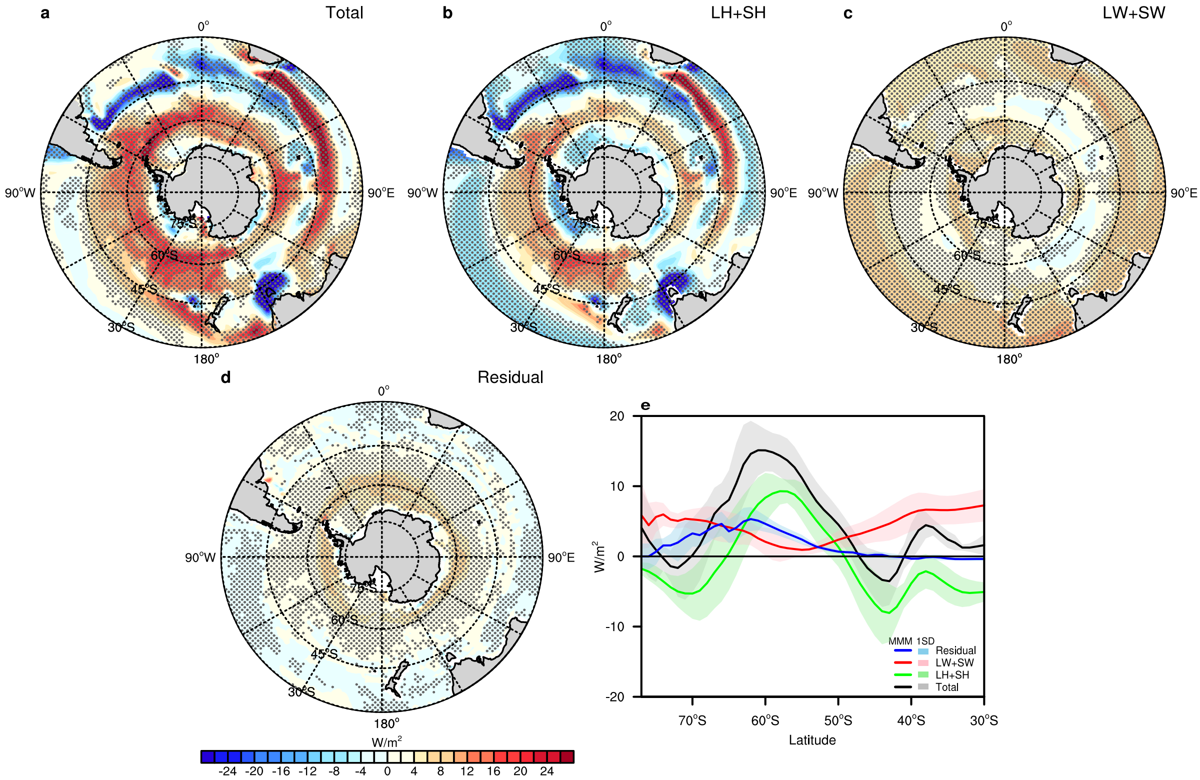

Supplementary Fig. 1 | Southern Ocean surface heat flux change from the historical to Shared Socioeconomic Pathway 5–8.5 (SSP585) scenario. a Difference of annual mean surface heat flux, and its contributions from **b** latent and sensible heat fluxes, **c** longwave and shortwave radiation fluxes, and **d** a residual flux term (shaded, units: W/m^2) for the multi-model mean (MMM) between CMIP5/6 SSP585 and historical simulations over the Southern Ocean. **e** Difference of annual and zonal mean surface heat flux contributed from the total (MMM, black; inter-model spread, gray), latent and sensible heat fluxes (MMM, green; inter-model spread, light green), longwave and shortwave radiation fluxes (MMM, red; inter-model spread, light red), and the residual (MMM, blue; inter-model spread, light blue) that includes heat fluxes due to frazil ice formation/melt for the MMMs between CMIP5/6 SSP585 and historical simulations over the Southern Ocean. The stipples refer to the regions where at least two-thirds of the models agree with the sign of the difference in the multi-model mean.

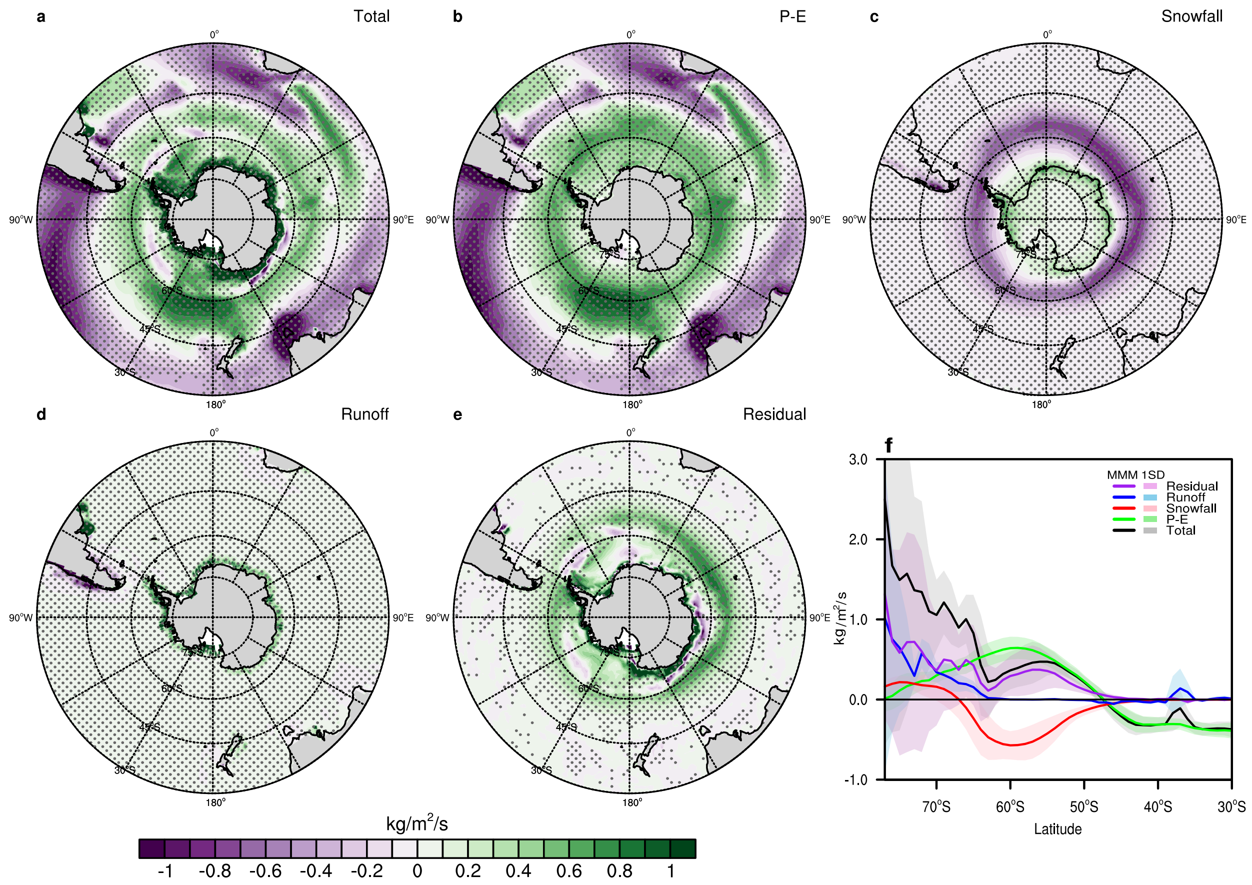

Supplementary Fig. 2 | Southern Ocean surface freshwater flux change from the historical to Shared Socioeconomic Pathway 5–8.5 (SSP585) scenario. a Difference of annual mean surface freshwater flux, and its contributions from **b** precipitation minus evaporation, **c** snowfall, **d** runoff that includes meltwater runoff from Antarctica, and **e** a residual term that includes sea ice melt (shaded, units: kg/m²/s) for the multi-model mean (MMM) between CMIP5/6 SSP585 and historical simulations over the Southern Ocean (SSP585 minus historical). **e** Difference of annual and zonal mean surface freshwater flux contributed from the total (MMM, black; inter-model spread, gray), precipitation minus evaporation (MMM, green; inter-model spread, light green), snowfall (MMM, red; inter-model spread, light red), runoff (MMM, blue; inter-model spread, light blue) that includes river runoff as well as meltwater from icebergs and ice shelves, and the residual (MMM, purple; inter-model spread, light purple) that includes sea ice melt and brine rejection for the MMMs between CMIP5/6 SSP585 and historical simulations over the Southern Ocean (SSP585 minus historical). The stipples refer to the regions where at least two-thirds of the models agree with the sign of the difference in the multi-model mean.

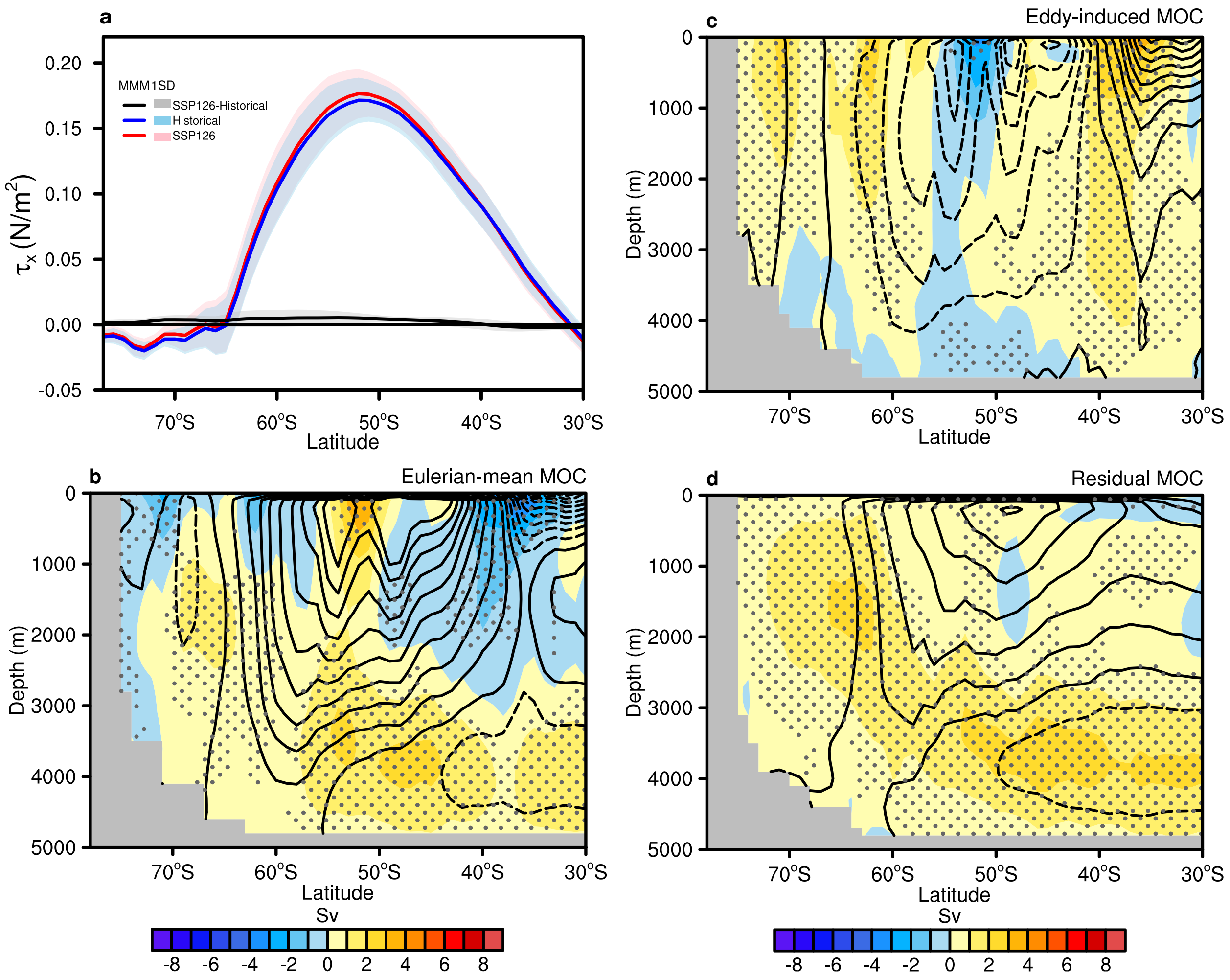

Supplementary Fig. 3 | Changes in zonal wind stress and Southern Ocean meridional overturning circulations (MOCs) from the historical to Shared Socioeconomic Pathway 1–2.6 (SSP126) scenario. a Annual and zonal mean surface zonal wind stress in the SSP126 (multi-model mean or MMM, blue; inter-model spread, light blue), historical (MMM, blue; inter-model spread, light blue) simulations, and their difference (SSP126 minus historical, MMM, black; inter-model spread, gray) over the Southern Ocean. The inter-model spread is defined as one standard deviation among models. **b** Difference in annual mean Southern Ocean Eulerian-mean MOC (shaded, units: Sv) for the MMM between SSP126 and historical simulations, overlaid with historical Eulerian-mean MOC annual mean climatology (contours, units: Sv). **c** same as **b**, but for the eddy-induced MOC. **d** same as **b**, but for the residual MOC. The stipples refer to the regions where at least two-thirds of the models agree with the sign of the difference in the multi-model mean.

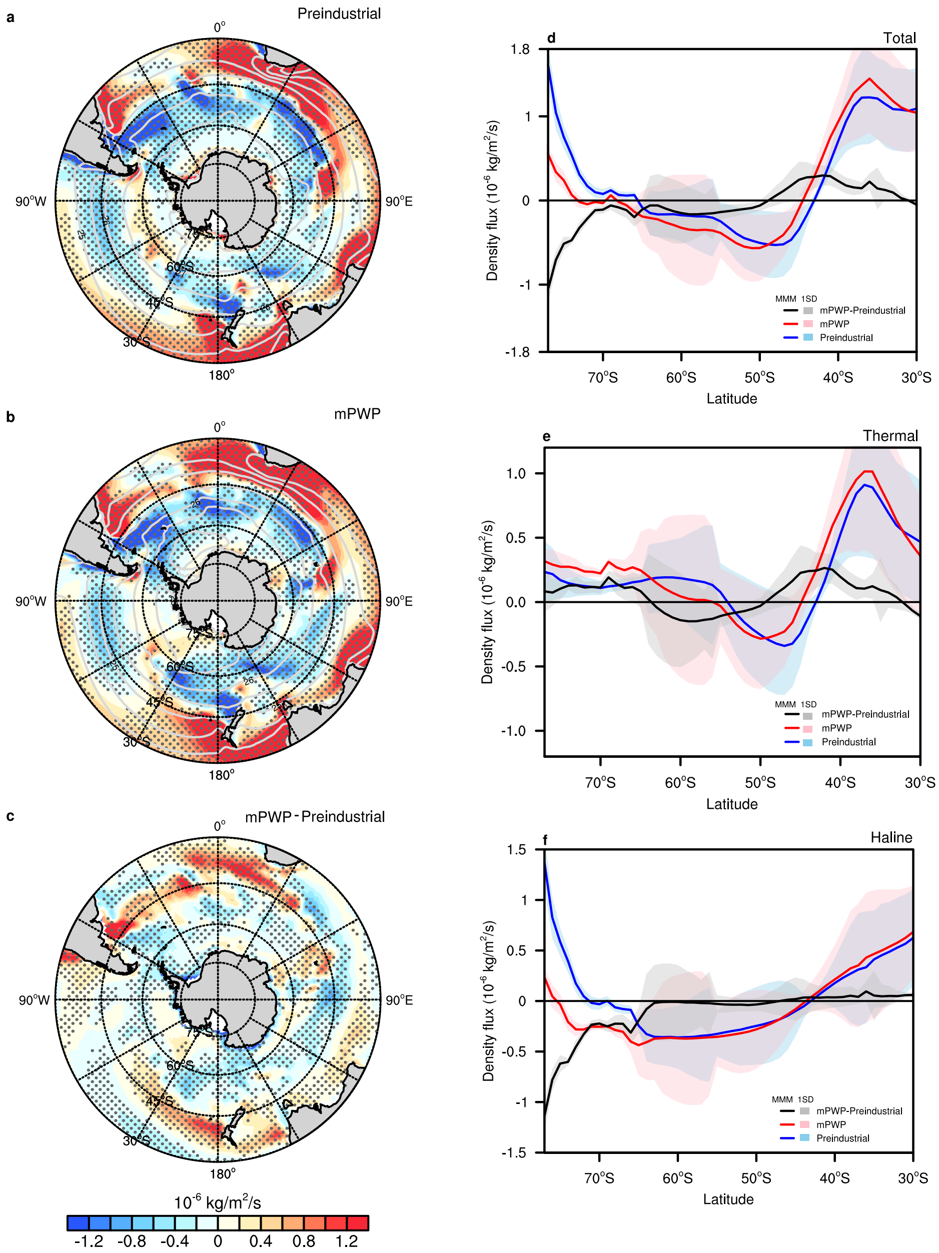

Supplementary Fig. 4 | Southern Ocean surface density flux during the preindustrial time and mid-Pliocene Warm Period (mPWP). a, b Annual mean surface density fluxes (shaded, unit: 10^{-6} kg/m²/s) and surface potential density (gray contour, unit: kg/m^3) for the multi-model means (MMMs) in CMIP5/6 **a** preindustrial and **b** mPWP simulations, as well as **c** the density flux difference between the two (mPWP minus preindustrial, shaded, unit: 10^{-6} kg/m²/s) over the Southern Ocean. **d** Annual and zonal mean surface density flux in preindustrial (MMM, blue; inter-model spread, light blue) and mPWP (MMM, red; inter-model spread, light red) simulations, and their difference (mPWP minus preindustrial, MMM, black; inter-model spread, gray). **e**, **f** Same as **d** but for the thermal and haline contributions to density flux, respectively. The stipples refer to the regions where at least two-thirds of the models agree with the sign of the multi-model mean.

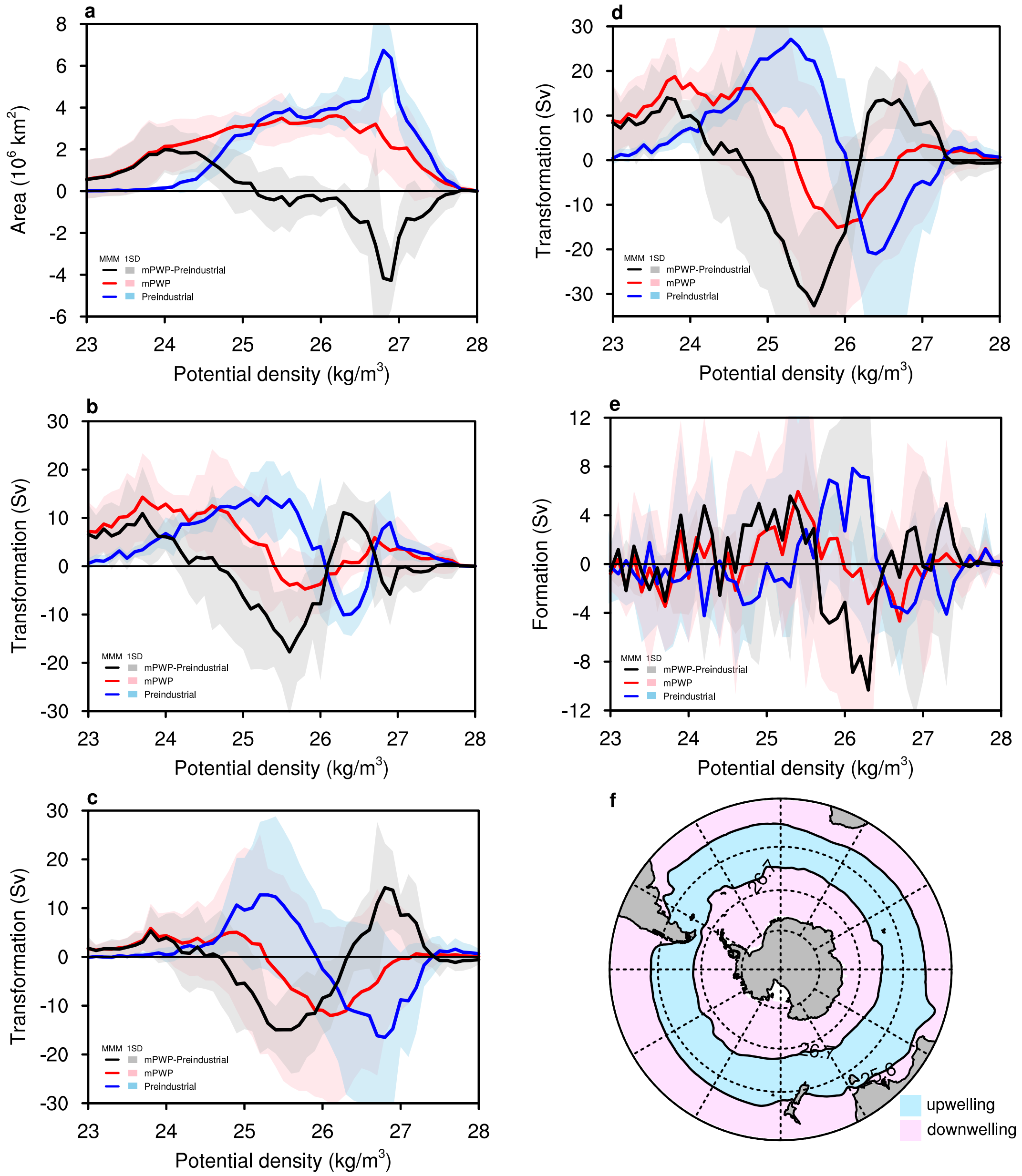

Supplementary Fig. 5 | Southern Ocean potential density area, water mass transformation, and formation during the mPWP and preindustrial time. a Area of potential density in CMIP5/6 mPWP (MMM, red; inter-model spread, light red) and preindustrial (MMM, blue; inter-model spread, light blue) simulations, and their difference (mPWP minus preindustrial, MMM, black; inter-model spread, gray) over the Southern Ocean. **b** Water mass transformation due to surface heat flux in mPWP (MMM, red; inter-model spread, light red) and preindustrial (MMM, blue; inter-model spread, light blue) simulations, and their difference (MMM, black; inter-model spread, gray). **c** Same as **b** but water mass transformation due to surface freshwater flux. **d** Same as **b** but the total water mass transformation with combined thermal and haline effects. **e** Water mass formation in mPWP (MMM, red; inter-model spread, light red) and preindustrial (MMM, blue; inter-model spread, light blue) simulations, and their difference (mPWP minus preindustrial, MMM, black, inter-model spread, gray). **f** Downwelling and upwelling regions, as indicated in **e**.

Supplementary Tables

Model	CMIP5/6	Historical	SSP585	SSP126
ACCESS-CM2	CMIP6	r(1,4,5,8)i1p1f1	r(2-5)i1p1f1	r(1-10)i1p1f1
ACCESS-ESM1-5	CMIP6	r(1-10)i1p1f1	r(1-10)i1pif1	r(1-10)i1p1f1
CESM1-CAM4*	CMIP5	r(1-6)i1pif1	r(1-6)i1pif1	
CESM1-CAM5*	CMIP5	r(1-3)i1pif1	r(1-3)i1pif1	
CESM2	CMIP6	r(4,10)i1p1f1	r(4,10)i1p1f1	r(4,10)i1p1f1
CMCC-CM2-SR5	CMIP6	r1i1p1f1	r1i1p1f1	r1i1p1f1
CMCC-ESM2	CMIP6	r1i1p1f1	r1i1p1f1	r1i1p1f1
CNRM-CM6-1	CMIP6	r(1-6)i1p1f2	r(1-6)i1p1f2	r(1-6)i1p1f2
CNRM-ESM2-1	CMIP6	r(1-10)i1p1f2	r(1-5)i1p1f2	r(1-5)i1p1f2
CanESM5	CMIP6	r(1-10)i1p1f1	r(1-10)i1p1f1	r(1-10)i1p1f1
FGOALS-g3	CMIP6	r(1-3)i1p1f1	r(1-4)i1p1f1	r(1-3)i1p1f1
FGOALS-f3-l	CMIP6	r(2-3)i1p1f1	r(1-2)i1p1f1	r(2-3)i1p1f1
HadGEM3-GC31-LL	CMIP6	r(1-5)i1p1f1	r(1-4)i1p1f1	r1i1p1f3
HadGEM-GC31-MM	CMIP6	r(2-4)i1p1f3	r(1-4)i1p1f1	r1i1p1f3
MPI-ESM1-2-LR	CMIP6	r(1-10)i1p1f1	r(1-10)i1p1f1	r(1-4,6-9)i1p1f1
MPI-ESM-1-2-HR	CMIP6	r(1-10)i1p1f1	r(1-2)i1p1f1	r(1-2)i1p1f1
MIROC6	CMIP6	r(1-10)i1p1f1	r(1-2)i1p1f1	r(1-2)i1p1f1
MRI-ESM2-0	CMIP6	r(1-2,4-10)i1p1f1	r(1-5)i1p1f1	r(1,2,4,5)i1p1f1
UKESM1-0-LL	CMIP6	r(1-4,8-10)i1p1f2	r(1-4,8)i1p1f2	r(1-4,8-10)i1p1f2

Supplementary Table 1. CMIP5/6 models, historical and future climate change simulations, and ensembles. The CMIP5/6 models and their ensemble historical, SSP585 and SSP126 simulation used in the current study. Models marked with * whose RCP85 simulations are used in the analysis as a substitute for SSP585.

Model	CMIP5/CMIP6	Preindustrial	mPWP
CESM2	CMIP6	r1p1i1f1	r1p1i1f1
CESM1-CAM4*	CMIP5	r1p1i1f1	r1p1i1f1
CESM1-CAM5*	CMIP5	r1p1i1f1	r1p1i1f1
ES-EARTH3-LR	CMIP6	r1p1i1f1	r1p1i1f1
GISS-E2-1-G	CMIP6	r1p1i1f1	r1p1i1f1
IPSL-CM6A-LR*	CMIP6	r1p1i1f1	r1p1i1f1

Supplementary Table 2. CMIP5/6 models and their preindustrial and mPWP simulations. The CMIP5/6 models as well as their preindustrial control and mPWP simulations used in the current study. Models marked with * have ideal age available in the outputs from both preindustrial and mPWP simulations.
